# Exploration of hypoglycemic peptides from porcine collagen based on network pharmacology and molecular docking

**DOI:** 10.1371/journal.pone.0298674

**Published:** 2024-03-12

**Authors:** Fating Zhou, Di Li, Yakun Hou, Zhihui Cong, Kaifeng Li, Xin Gu, Guosheng Xiao

**Affiliations:** 1 College of Biology and Food Engineering, Chongqing Three Gorges University, Chongqing, China; 2 College of Food Science and Technology, Hebei Agricultural University, Baoding, China; The Islamia University of Bahawalpur Pakistan, PAKISTAN

## Abstract

In recent years, the extraction of hypoglycemic peptides from food proteins has gained increasing attention. Neuropeptides, hormone peptides, antimicrobial peptides, immune peptides, antioxidant peptides, hypoglycemic peptides and antihypertensive peptides have become research hotspots. In this study, bioinformatic methods were used to screen and predict the properties of pig collagen-derived hypoglycemic peptides, and their inhibitory effects on α-glucosidase were determined *in vitro*. Two peptides (RL and NWYR) were found to exhibit good water solubility, adequate ADMET (absorption, distribution, metabolism, elimination, and toxicity) properties, potentially high biological activity, and non-toxic. After synthesizing these peptides, NWYR showed the best inhibitory effect on α-glucosidase with IC_50_ = 0.200±0.040 mg/mL, and it can regulate a variety of biological processes, play a variety of molecular functions in different cellular components, and play a hypoglycemic role by participating in diabetic cardiomyopathy and IL-17 signaling pathway. Molecular docking results showed that NWYR had the best binding effect with the core target DPP4 (4n8d), with binding energy of -8.8 kcal/mol. NWYR mainly bonded with the target protein through hydrogen bonding, and bound with various amino acid residues such as Asp-729, Gln-731, Leu-765, etc., thus affecting the role of the target in each pathway. It is the best core target for adjuvant treatment of T2DM. In short, NWYR has the potential to reduce type 2 diabetes, providing a basis for further research or food applications as well as improved utilization of pig by-products. However, in subsequent studies, it is necessary to further verify the hypoglycemic ability of porcine collagen active peptide (NWYR), and explore the hypoglycemic mechanism of NWYR from multiple perspectives such as key target genes, protein expression levels and differences in metabolites in animal models of hyperglycemia, which will provide further theoretical support for its improvement in the treatment of T2DM.

## 1. Introduction

During animal processing, nearly 40% of the carcass is considered a by-product. Statistics show that the annual output of pork was >49 million tonnes in China, producing nearly 5 million tonnes of viscera, 7 million tonnes of skins, and 8 million tonnes of bones [1]. These by-products contain a lot of protein, with collagen being the most abundant. Converting these by-products into collagen, collagen peptide, etc., can increase its utilization value. The global collagen market is estimated to be worth around US $7 billion by 2027 [2]. Porcine collagen peptide is similar in structure to human collagen peptide and generally does not cause an allergic response, and contains a large amount of protein, vitamins, calcium, iron, phosphorus and other nutrients, with nutritional supplement, provide energy, promote bone growth, help improve anemia and skin conditions and other effects and effects. With the development of high technology such as combinatorial biotechnology, the high-value utilization of low molecular bioactive peptides has become a research hotspot. At present, a variety of bioactive peptides of pig collagen have been identified from pig by-products by bioinformatics combined with network pharmacology, which provides an opportunity for high-value utilization of animal by-products [3, 4]. Diabetes is a chronic, non-infectious condition that can cause a serious threat to global public health [5]. The most recent data available from the International Diabetes Federation (IDF) in 2021 indicate that the global prevalence of diabetes among people aged 20 to 79 is estimated at 10.5% (about 537 million people) and is expected to increase to 12.2% (783.2 million people) by 2045 [6]. Clinical drugs, such as sulfonylureas and biguanides, can be used to control blood glucose levels in T2DM, but they also induce a series of side effects, including diarrhea, liver damage, and abdominal distention, and drug resistance can occur [7, 8]. In addition, long-term and high-dose use of synthetic drugs is severely restricted because of the potential health-related risks. It has become an inevitable trend for people to search for low toxicity, low price and effective natural active substances from natural resources to replace synthetic drugs in the treatment of type 2 diabetes [9, 10]. α-Glucosidase can directly participate in the metabolic pathway of starch and glycogen, regulate the human sugar chemical metabolism, and is an indispensable enzyme in the biological glucose metabolism pathway. Therefore, the search for novel α-glucosidase inhibitory peptides is of great significance because of their potential as components of biopharmaceuticals or nutraceuticals to alleviate diabetes-related health burdens.

Bioactive peptides (BPs) are generated from diversified protein resources by enzymatic hydrolysis, chemical degradation and microbial fermentation methods [11]. In addition to their widely accepted nutritive value, BPs also have important biological activities, including anti-diabetes, antioxidant, antihypertensive, anti-inflammatory, hypolipidemic, immunomodulatory and mineral binding, and thus are of great value in promoting human health [11–13]. Then, BPs draw great interest from consumers and have been applied in a wide variety of products, especially functional food, daily cosmetics, medicinal/pharmaceutical products, and nutritional supplements [13]. Food components, such as proteins and peptides, are currently of great interest because of their potential roles in the prevention and control of diabetes through blood sugar regulation [14–17]. Recent studies have highlighted the potential of collagen from pig skin during slaughtering and processing as a hypoglycemic peptide due to its high hydroxyproline (Hyp), proline (Pro), and glycine (Gly) content [18]. It has a variety of beneficial effects in the control of diabetes, including improving hyperglycemia, reducing fasting blood glucose levels, increasing glucose tolerance, mitigating excessive thirst and hunger symptoms, improving liver and kidney functions, reducing free radical formation due to glucose self-oxidation, and decreasing protein glycosylation [19]. Moreover, collagen peptide has shown promise in reducing the number of physiological reactions that lead to insulin resistance (IR), including modulating free fatty acid levels, leptin, and resistin, and preventing the resulting increase in the incidence of T2DM and cardiovascular disease, thus averting complications of diabetes [20]. Clinical studies have found that collagen peptide supplementation can improve insulin secretion function and cord sensitivity, improve glucose load after glucose area under the curve, meaningfully shorten the blood glucose adjustment period, and reduce the incidence of nocturnal hypoglycemia in diabetic patients. Additionally, it can improve the nutritional status and immunity of patients after surgery, as well as shorten the average hospital stay of patients after surgery [21].

At present, dietary proteins isolated from black beans, fermented soybean, balsam pear, and other sources have been identified as having anti-diabetic peptide properties [22, 23]. *In vitro* experiments have confirmed the hypoglycemic effects of many of these peptides, with some outperforming clinical drugs [24]. Specifically, *Zhang*, *Y et al*. identified four peptides from silkworm pupae: NSPR, QPPT, SQSPA, and QPGR, which can suppress α-glucosidase activity and help manage diabetes [25]. *He L*, *et al*. isolated five peptides (GPVGPPG, GPPGPT, APGGAP, FGPGP, and GPVG) from bovine skin collagen, and all of which exhibited anti-diabetic properties [26]. The discovery of natural active ingredients for treating T2DM has become a research hotspot due to their safety, reduced or no side effects, and efficient absorption [25].

Network pharmacology is an efficient approach to predicting the underlying mechanisms of disease-drug interactions based on systems biology and the integration of various technologies such as network and pharmacology analysis [27]. In recent years, network pharmacology has been widely used to predict the mechanism of action between active ingredients and disease. Pan et al. [28] revealed the hypoglycemic mechanism of aloe emodin through network pharmacology, and the results showed that aloe emodin has 22 core targets for improving the treatment of T2DM. Such as serine/Threonine-protein kinase-1 (AKT1), mitogen activated protein kinase 8 (MAPK8), etc. These targets are mainly concentrated in signaling pathways such as PI3K-Akt and insulin resistance. Zhou et al. [29] studied the role of GPPGPA, a peptide screened from the skin collagen hydrolysate of Salamandus chinanalis, in T2DM and related molecular mechanisms, and identified the core targets as AKT1, MAPK8, and transcription factor AP-1 (JUN) by network pharmacology. These targets mainly focus on the PI3K-Akt signaling pathway related to T2DM, AGE-RAGE signaling pathway in diabetic complications, tumor necrosis factor (TNF) signaling pathway, and insulin resistance. Tian et al. [30] studied the antibacterial active components and their mechanisms of action of radix isatis based on network pharmacology, and the results showed that radix isatis mainly regulates apoptosis-related cysteine peptidase (CASP3) through stigmosterol, prostaglandin-endoperoxide synthase 2 (PTGS2) and other targets, gene functions are enriched in cell apoptosis, transcriptional regulation, and participate in cancer pathway and TNF signaling pathway to play a antibacterial role.

Therefore, this study screened peptides through an online database, synthesized peptides with the highest activity, and further screened them through *in vitro* experiments. The potential mechanism of action of hypoglycemic peptides derived from porcine collagen was revealed through network pharmacology and molecular docking. The purpose of this research was to offer a new perspective on improving the utilization value of pig by-products, identifying food-derived hypoglycemic peptides, and establishing a theoretical foundation for understanding the machine-processed porcine collagen peptides’ multi-target and multi-channel treatment of type 2 diabetes.

## 2. Materials and methods

### 2.1 Materials

Peptides RL and NWYR were synthesized by Xi’an Na Microbiology Co., Ltd. pNPG (4-nitrobenzene-α-D-glucopyranose) and α-glucosidase were bought from Shanghai Yuanye Bio-Technology Co., Ltd. PBS (phosphate-buffered saline, formulated from KH_2_PO_4_ and NaH_2_PO_4_) and Na_2_CO_3_ were obtained from Chengdu Colon Chemical Co., Ltd. The microplate reader used was an iMark made in Japan, and the 37°C incubator used was a SPX-250F-III from Shanghai Longyue Instrument Equipment Co., Ltd.

### 2.2 Acquisition and activity evaluation of porcine collagen

The sequences of porcine collagens were obtained from the NCBI database (National Center for Biotechnology Information, http://www.ncbi.nlm.nih.gov/protein).The potential of porcine collagen to release α-glucosidase inhibitory peptides was evaluated using BIOPEP-UWM [31] (https://biochemia.uwm.edu.pl/biopep-uwm/). The calculation of the α-glucosidase inhibitory peptide (A) release quantity is as follows:

$$A\%=\frac{a}{N}\times 100\%$$ (1)

where a is the amount of α-glucosidase inhibiting peptide fragments in the protein sequence, and N is the length of the protein sequence.

### 2.3 *In silico* digestion analysis and virtual screening

The theoretical peptide sequence was obtained by virtual enzymatic hydrolysis of the screened porcine collagen with pepsin (EC3.4.23.1) and trypsin (EC3.4.21.4) in ExPASy PeptideCutter [32] (https://www.expasy.org/resources/peptidecutter) program. The activity fractions of released dipeptides, tripeptides, and tetrapeptids were numerated by PeptideRanker (http://distilldeep.ucd.ie/PeptideRanker/). Generally, peptides with an activity score >0.5 were considered prospective bioactive peptides, and the tool available online at Innovagen was used to predict water solubility [33] (http://www.innovagen.com/proteomics-tools). The BBB (blood brain barrier), HIA (human intestinal absorption), absorption, distribution, metabolism, elimination, and toxicity (ADMET) properties, including toxicity, metabolism, and Caco-2 permeability, were analyzed in admetSAR (http://lmmd.ecust.edu.cn/admetsar1/) [34]. To forecast the potential toxicity of the identified peptides, the online tool ToxinPred (https://webs.iiitd.edu.in/raghava/toxinpred/index.html) was used.

### 2.4 Peptide synthesis

The purity, amino acid composition, and molecular weight of the peptides were provided by the manufacturer. The peptides that were screened *in silico* were synthesized using solid-phase synthesis by Xi’an Na Microbiology Co., Ltd, and their α-glucosidase inhibitory activity was tested *in vitro*.

### 2.5 α-Glucosidase activity inhibition test

The α-glucosidase test was conducted based on Ramadhan’s method with minor modifications [35]. The samples were prepared at different concentrations (1–7 mg/mL), and the following compounds were prepared: α-glucosidase (0.1 Ua), PBS (pH 6.8, 0.05 mol/L), PNPG (2.5 mmol/L), and Na_2_CO_3_ (1 mol/L). The control, sample blank, and sample groups were incubated at their appropriate temperature and time, and the absorbance at 405 nm was measured using a microplate reader. The inhibitory activity was calculated using Eq (2). The inhibition rate of α-glucosidase was solved according to the concentration of different mass samples. The IC_50_ values were calculated based on the fitting curves.

$$Inhibition\%=1-\left(\frac{A_{0}-A_{1}}{B_{0}-B_{1}}\right)\times 100\%$$ (2)

where A_0_, A_1_, B_0_, and B_1_ are the absorbance of sample, blank, control, and sample blank, respectively.

### 2.6 Network pharmacology analysis

#### 2.6.1 Target prediction

The molecular structure and SMILES format of the peptides were confirmed using PepDraw (http://pepdraw.com/) and NovoPro (https://www.novopro.cn/tools/), respectively. The target gene associated with the selected peptide was appraised utilizing the SwissTargetPrediction data bank (http://www.swisstargetprediction.ch/) with "Homo sapiens" as the selected species (probability ≥0.1). Using "type 2 diabetes" and "T2DM" as the operative words, targets associated with type 2 diabetes were screened from the OMIM data bank (http://www.omim.org), the human genome database GeneCards (https://www.genecards.org/), and the TTD data bank (http://bidd.nus.edu.sg/group/cjttd) [29].

#### 2.6.2 Construction of protein-protein interaction network

Target peptide-related target genes and type 2 diabetes targets were plotted by Venny 2.1.0 (https://bioinfogp.cnb.csic.es/tools/venny/index.html) and common targets were derived. These were then investigated as potential therapeutic peptide targets in T2DM treatment. The identified common targets were uploaded to the STRING database [36] (http://string-db.org/), with "Homo sapiens" picked out as the species and the minimum interaction threshold set to "medium confidence (0.4)". The resulting protein-protein interaction (PPI) network was analyzed using Cytoscape 3.9.1 (http://www.cytoscape.org/) and visualized to gain further insights.

#### 2.6.3 KEGG and GO pathway enrichment analysis

The collective targets related to target peptides and type 2 diabetes were imported into the Metscape platform (https://metascape.org/gp/index.html), and KEGG (kyoto encyclopedia of genes and genomes) pathway analysis and GO (gene ontology) analysis were performed with a P-value of ≤ 0.01 as the threshold [37]. Finally, the obtained data are visualized and analyzed by the bioinformatics platform.

### 2.7 Molecular docking

The data including grid sizes, ligand (. pdbqt) and protein files were utilized for simulation, followed by further processing using AutoDock Tools 1.5.6, which is devised to be compatible with .pdbqt format. The PDB (Protein Data Bank; http://www.rcsb.org) is an archive of 3D structural data of biological macromolecules, such as complex assemblies, nucleic acids, and proteins [38]. The key target was selected, and the corresponding protein with a higher resolution was found in the PDB. The 3D structural formula was downloaded, and water molecules and hydrogenated proteins were removed by Autodock Tools 1.5.6 software. The key targets and screened peptide structures were imported into Autodock Vina for molecular docking verification tests. Finally, the 3D schematic diagram was constructed using PyMol 2.5.2 software.

### 2.8 Statistical analysis

All experiments were conducted in triplicate and the data are expressed as the mean ± standard deviation (SD). Statistical analysis was performed using SPSS 27.0 software with Duncan’s multiple and one-way ANOVA tests to determine the differences among the mean values. The mapping was done using prism and bioinformatics tools (http://www.bioinformatics.com.cn/).

## 3. Results

### 3.1 Subsection

A search for collagen in the NCBI database, with the species set to “Sus scrofa domesticus”, the chain structure of collagen type-Ⅰ was obtained, specifically the α-1 chain and α-2 chain with accession numbers of BAX02568.1 GI: 1159729721 and BAX02569.1 GI: 1159729723, respectively. The lengths of the chains were 1466 aa and 1366 aa, respectively. Moreover, the BIOPEP-UWM predicted that the theoretical release amounts of the two α-glucosidase inhibitory peptides were 5.2% and 4.1%, and their theoretical fragments were 76 and 57, respectively. Thus, both porcine collagen chains had the potential to release α-glucosidase inhibitory peptides.

### 3.2 *In silico* identification of α-glucosidase inhibitory peptides in porcine collagen proteins

FASTA formats of the α-1 and α-2 chain structures were retrieved from NCBI and copied into PeptideCutter for collagen hydrolysis. Two gastrointestinal hydrolases, pepsin and trypsin, were selected to hydrolyze porcine collagen and evaluate potential α-glucosidase inhibitory peptides [3]. Since their versatile cleavage sites, these enzymes have also been applied in food industry. The two porcine collagen chains were enzymatically hydrolyzed, and di-, tri-, and tetrapeptides were collected. After removing repetitive peptides, a total of 54 oligopeptides were screened, and their water solubility and biological activities were predicted. In general, bioactive peptides are defined as those with a PeptideRanker score greater than 0.5 on a scale of 0 to 1, with a higher value indicating a higher likelihood of being biologically active [32, 33]. To reduce false positive scores, peptides with a threshold value ≥ 0.6 were selected. As a result, a total of five peptide sequences with good water solubility and potential biological activity were obtained, as shown in Table 1.

**Table 1:** The results of virtual screening analysis of the selected peptides.

Peptide sequence	Activity score	Water solubility	BBB	HIA	Toxicity	Protein sources (Accession)	Positions of cleavage sites	Name of cleaving enzyme(s)
GPR	0.87	Good	BBB- (0.69)	HIA- (0.67)	None	BAX02568.1 BAX02569.1	126, 912 41, 822, 1017	Trypsin
MRL	0.82	Good	BBB- (0.63)	HIA+ (0.64)	None	BAX02569.1	1259	Pepsin (pH 1.3)
NWYR	0.82	Good	BBB+ (0.50)	HIA+ (0.85)	None	BAX02569.1	1218	Trypsin
GR	0.77	Good	BBB+ (0.76)	HIA- (0.52)	None	BAX02568.1 BAX02569.1	224 1172	Trypsin
RL	0.63	Good	BBB+ (0.70)	HIA+ (0.83)	None	BAX02568.1	9, 1359	Pepsin (pH 1.3)

To predict the ADMET properties of these five peptides in admetSAR, the Molecular Linear Input specification (SMILES) format of the peptides was simplified by NovoPro online tool. Peptide- and protein-based drugs have gained increasing interest; however, their unknown toxicity has significantly limited development. The toxicity of bioactive peptides has become a major concern in the development of peptide healthcare products. To investigate the toxicity of collagen peptides, the *in silico* tool ToxinPred has been applied, which can predict the toxicity of collagen peptides. The ADMET prediction and toxicity results are shown in Table 1. Out of the five peptides screened, only NWYR and RL demonstrated acceptable ADMET properties and were HIA+ and BBB+, indicating that they are easily absorbed and can pass through the blood-brain barrier. These peptides were also identified with few or no side effects, making them suitable for use in medicine and food. Therefore, RL and NWYR, with good water solubility, permissible ADMET properties, and a bioactivity fraction greater than 0.6, were selected for synthesis and *in vitro* α-glucosidase activity inhibition testing.

### 3.3 Inhibitory activity of RL and NWYR on α-glucosidase

**Fig 1:**
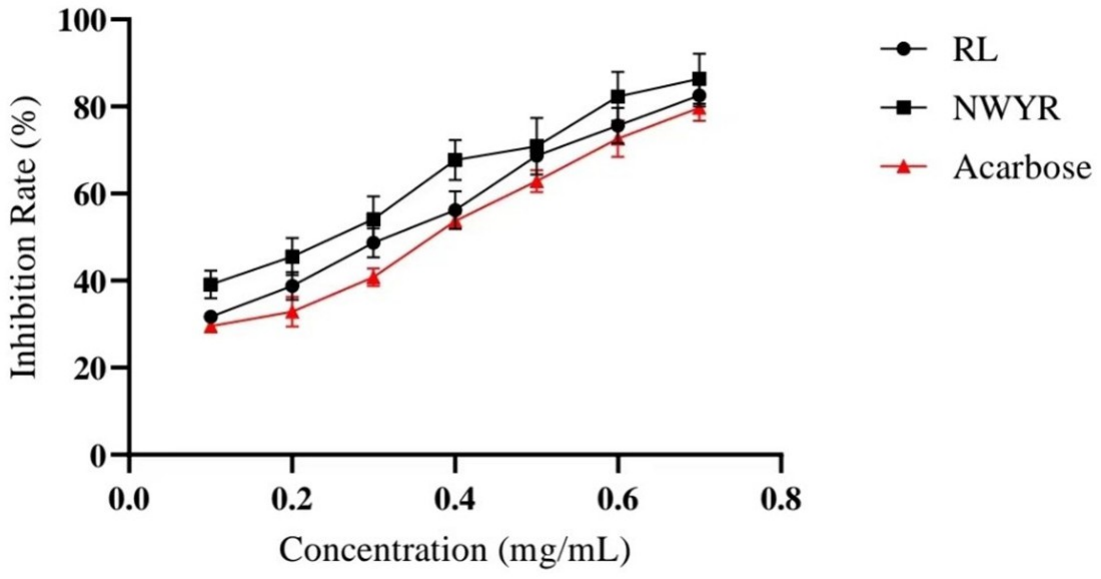
Inhibitory effect of RL and NWYR on α-glucosidase.

As shown in Fig 1, RL and NWYR significantly inhibited α-glucosidase activity in a concentration-dependent manner *in vitro*. The inhibitory effect of RL and NWYR on α-glucosidase activity increased with increasing concentration from 0.1 to 0.7 mg/mL. By inhibiting curve calculation, the IC_50_ values of α-glucosidase for tetrapeptide (NWYR) and dipeptide (RL) were 0.200 ± 0.040 mg/mL and 0.264 ± 0.005 mg/mL, separately. These data were slightly lower than that of acarbose (IC_50_ = 0.346 ± 0.043 mg/mL) (Table 2).

**Table 2:** Linear equations and IC_50_ values of compounds and control.

Compound	Linear equation	IC_50_ value
Peptide RL	y = 87.821x + 22.333	0.264 ± 0.005 mg/mL
Peptide NWYR	y = 82.964x + 30.529	0.200 ± 0.040 mg/mL
Control Acarbose	y = 90.119x + 17.11	0.346 ± 0.043 mg/mL

### 3.4 Common and key targets of NWYR-T2DM

**Fig 2:**
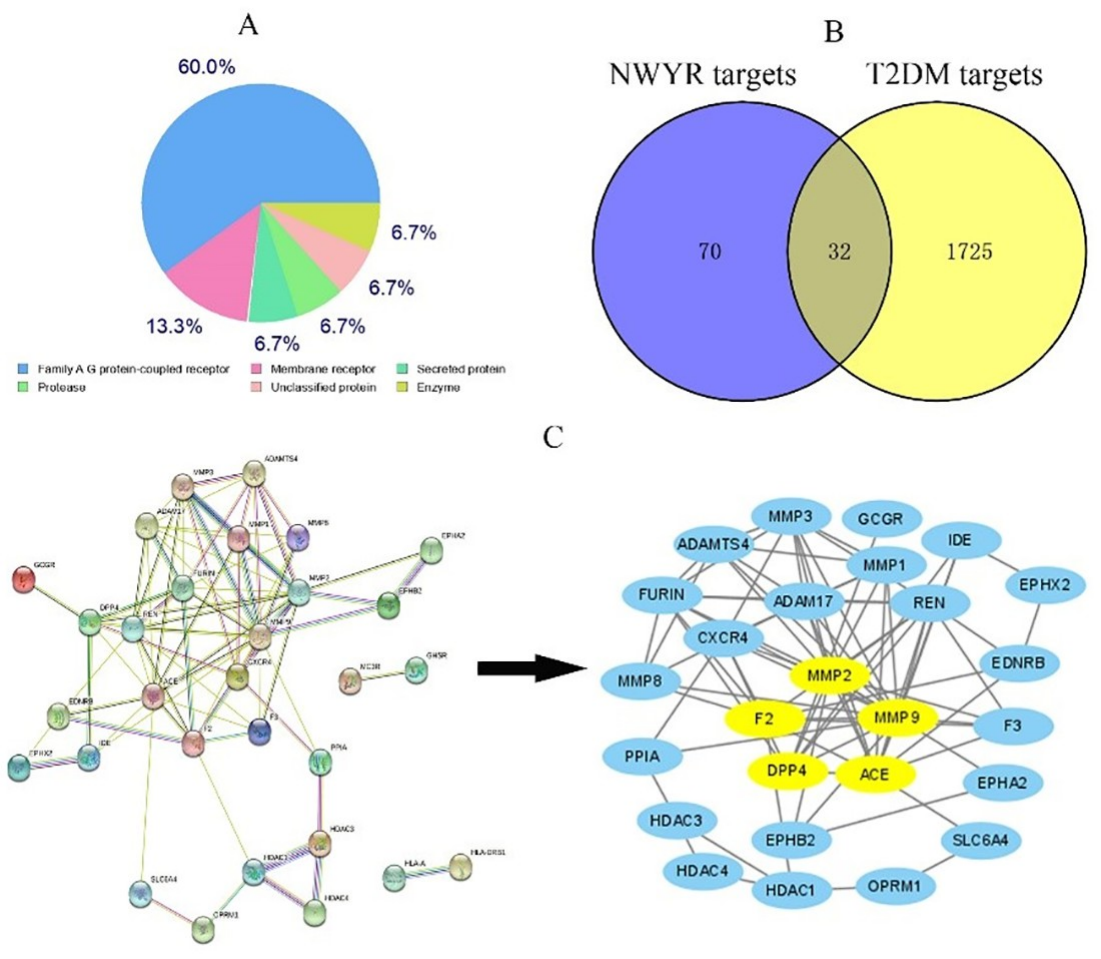
A: Classification of proteins that may theoretically interact with NWYR; B: The intersection of NWYR and T2DM targets; C: PPI network of common targets of NWYR and T2DM ((the yellow targets are key nodes according to topological analysis)).

The 3D structure of NWYR was uploaded to the SwissTargetPrediction website to predict peptide targets. After eliminating duplicates, 100 related targets were displayed. SwissTargetPrediction is a program used to support new drug design and discovery. It provides protein classification of potential targets of small molecules (Fig 2A). Among the potential proteins that can interact with the peptides, the most abundant was the Family A G protein-coupled receptor, accounting for 60.0%. A search based on the keyword "T2DM" in the GeneCards database yielded 1215 relevant targets, and 614 targets associated with T2DM were gathered from the OMIM and TTD databases. To complement relevant targets and remove duplicates, 1762 targets associated with T2DM were obtained. The online tool Venny 2.1.0 was used to draw the interactive network of NWYR and T2DM targets (Fig 2B). After the intersection, 32 common targets of NWYR-T2DM were obtained. These collective targets were then imported into STRING to draw a PPI network, and two disconnected nodes in the network were removed (Fig 2C). Topology analysis of the network was conducted using the Centiscape 2.2 plug-in in Cytoscape 3.9.1. Screening parameters (Degree = 5.05, Betweenness = 29.154, Closeness = 0.019) were set based on the calculated median, and five major targets were ultimately acquired (Table 3), accounting for 15.63%. These targets played critical roles in the whole network and were identified as significant targets for the treatment of T2DM.

**Table 3:** Key targets of NWYR-T2DM.

Key tagets (Gene name)	Betweenness	Closeness	Degree
ACE	103.764	0.025	12
MMP2	69.158	0.023	13
F2	105.769	0.023	9
MMP9	136.757	0.028	16
DPP4	55.771	0.0218	8

### 3.5 KEGG and GO enrichment results of NWYR-T2DM

**Fig 3:**
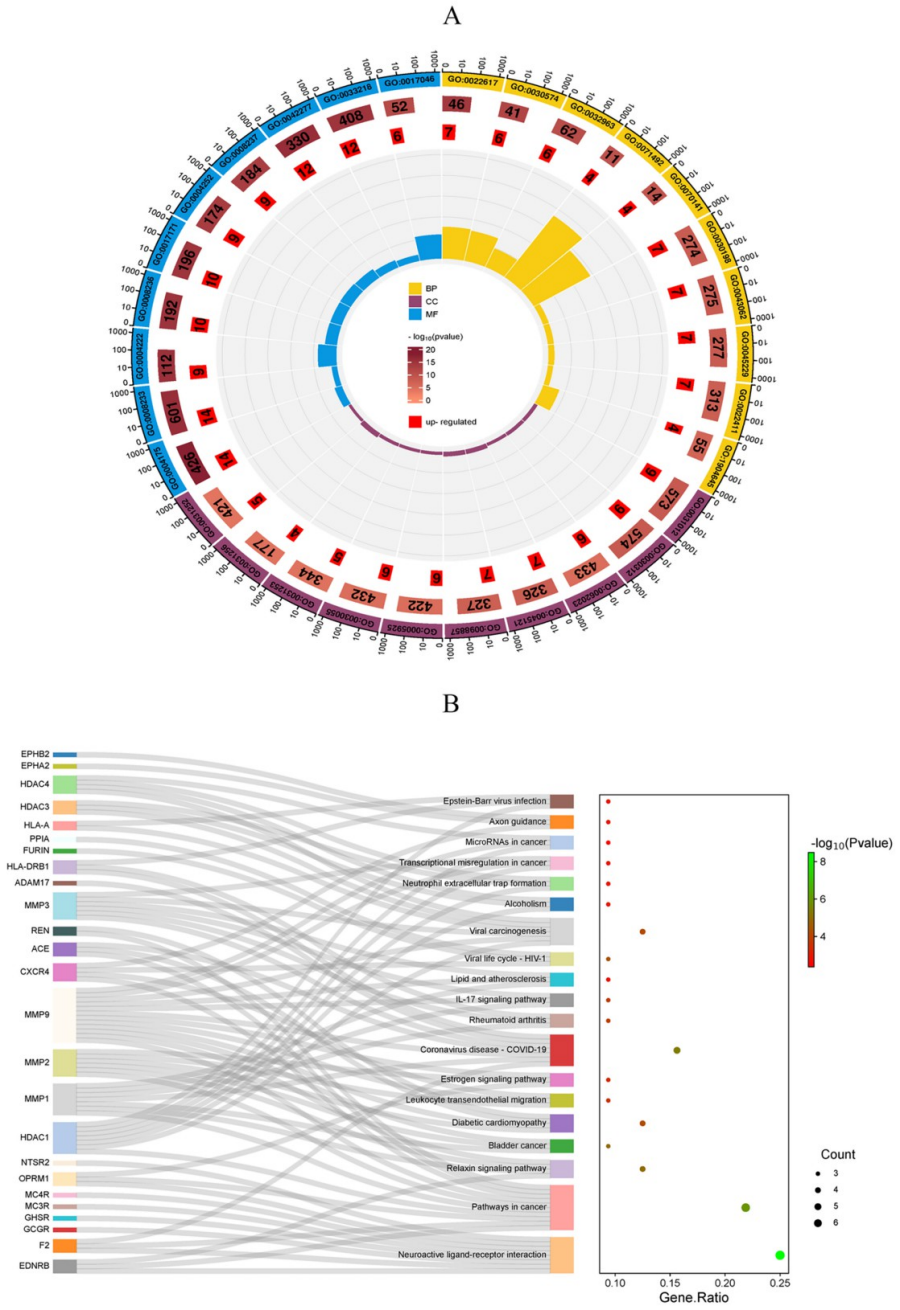
GO and KEGG enrichment analysis of NWYR-T2DM A: GO enrichment analysis of NWYR-T2DM. (Yellow: Biological Process; Purple: Cellular Component; Blue: Molecular Function.) B: KEGG pathway analysis of NWYR-T2DM.

Metscape database was used to perform KEGG and GO pathway enrichment analysis of the 32 common targets (*P* < 0.01). GO enrichment analysis produced a total of 388 records, with biological process (314), cell composition (39), and molecular function (35) accounting for 80.93%, 10.05%, and 9.02%, respectively. The top 10 results from the GO enrichment were used to generate a statistical histogram of enrichment (Fig 3A). Target proteins in the biological process category were mainly involved in reactions with lipopolysaccharides, response to bacteria, insulin, and oxygen; positive regulation of cell activation, death, growth, and motility; regulation of body fluid levels; cation transmembrane transport; and reactive oxygen species metabolic processes. The proteins of molecular functions were mainly related to hydrolase activity, endopeptidase activity, acting on carbon-nitrogen (but not peptide) bonds, G protein-coupled peptide receptor activity, peptide binding, and immune receptor activity. In the cellular components category, target proteins were predominantly found in the membrane raft, exosomes, and extracellular matrix. KEGG analysis revealed 19 signaling pathways, including the diabetic cardiomyopathy pathway and IL-17 signaling pathway that are involved in diabetic complications, neuroactive ligand-receptor interactions, bladder cancer, and transcriptional misregulation in cancer. A bubble chart was created using the pathways (Fig 3B).

### 3.6 Molecular docking results

To demonstrate their possible interaction mechanism, the central targets were docked with the screened active peptides. Based on the key targets selected in Section 3.4, the corresponding proteins were located in the PDB database and their 3D structure formulas were downloaded: ACE (7a2x), F2 (1e8b), MMP2 (7xjo), MMP9 (6esm), and DPP4 (4n8d). Molecular docking of these proteins with NWYR revealed that the binding energies of DPP4-NWYR, MMP2-NWYR, ACE-NWYR, F2-NWYR, and MMP9-NWYR were -8.8, -8.7, -8.5, -7.3, and -6.4 kcal/mol, respectively. The smaller the binding energy, the stronger and more stable the binding. Therefore, the top three were chosen for structure-activity analysis (Table 4).

**Table 4:** Docking results and binding free energy (kcal/mol) of peptides by virtual screening.

Small molecules(peptide) and receptor proteins	Binding energy (kcal/ mol)	Number of hydrogen bonds	Binding amino acids and sites
DPP4-NWYR	-8.8	8	Asp-729, Gln-731, Leu-765
MMP2-NWYR	-8.7	9	Asp-33, Arg-53, Ile-54, Tyr-55, Arg-19
ACE-NWYR	-8.5	7	Glu-98, Thr-130, Gly-128

## 4. Discussion

### 4.1 Silicon screening analysis

Trypsin cleaves proteins at the carboxy site of Lys and Arg, while pepsin targets C-terminal end Glu, Leu, or Phe. These enzymes have also been used to produce various structures of bioactive peptides [39, 40]. The bioactivity and function of peptides are closely correlated with their chain length and amino acid sequence. Bioactive peptides are better than individual amino acids (AAs) in clinical application because short peptide chains present lower osmotic pressure and higher intestinal absorption rates than those of the corresponding free Aas [41]. Besides, short chain peptides are more stable and easier to be absorbed *in vivo* [42]. Therefore, dipeptides, tripeptides, and tetrapeptides were selected for further screening of α-glucosidase inhibitory peptides. *In silico* drug design, the properties of ADMET and the drug-likeness of the molecules need to be predicted [43], and comparing the properties of different food components and drugs has become an increasingly popular research topic [44]. ADMET characterization can help *in silico* evaluation of potential peptide bioactivities. However, research on bioactive peptide ADMET properties in food are rarely reported [45]. This study primarily predicted the BBB and HIA properties, as HIA helps predict small intestine absorption and physiological barriers limit most compounds.

### 4.2 Effect of α-glucosidase on NWYR

α-Glucosidase, which is exist in the epithelium of the small intestine, is a membrane-bound glycoenzyme that facilitates glucose absorption by catalyzing the hydrolysis of oligosaccharides and disaccharides into absorbable monosaccharides. Suppressing α-glucosidase activity can attenuate digestion of carbohydrate, decrease the hydrolysis of oligosaccharides and polysaccharides into monosaccharides, thereby reducing blood glucose levels and alleviating diabetes [46, 47]. The results of α-glucosidase inhibition showed that both NWYR and RL had inhibitory effects on α-glucosidase activity, and there was no significant difference between them and acarbose, but the inhibitory effect of NWYR was stronger. The NWYR peptide sequences contain basic amino acids (arginine) and hydrophobic amino acids (tryptophan, tyrosine), which are consistent with the hypoglycemic peptides previously found in collagen. Based on this observation, we predict that NWYR will have a therapeutic effect on T2DM. Therefore, NWYR was selected for molecular docking and network pharmacology to investigate its mechanism of action on T2DM.

### 4.3 KEGG and GO enrichment analysis

GO enrichment analysis demonstrate that NWYR can regulate various biological processes and molecular functions in different cellular components to achieve its anti-diabetic effect. KEGG enrichment analysis showed that NWYR could play a hypoglycemic role through several signaling pathways, including the diabetic cardiomyopathy pathway and IL-17 signaling pathway that are involved in diabetic complications, neuroactive ligand-receptor interactions, bladder cancer, and transcriptional misregulation in cancer. Epidemiological studies have shown that diabetes is associated with an increased risk of cancer, and diabetic cardiomyopathy is one of the leading causes of death in patients with diabetes, especially type 2 diabetes. Studies have shown that the mechanism of IL-17 promoting diabetes is related to the inflammatory destruction of islet cells. *In vitro*, IL-17 induces SOD2 transcription and synergies with IL-1β and IFN-γ to promote the expression of NOS2A and COX-2 and the production of oxygen free radicals, enhancing the inflammatory response in islet cells. In addition, IL-17 can also inhibit the transcription of anti-apoptotic gene BCL-2 mRNA and accelerate the apoptosis of islet cells, which is closely related to the onset of diabetes. Blocking IL-17 signaling pathway is expected to become a new target for the treatment of diabetes. *Fang et al*. found that the treatment of type 2 diabetes is regulated through pathways in cancer signaling [48]. This suggests that NWYR could be used to treat T2DM and related complications via multiple pathways and targets.

### 4.4 Molecular docking analysis

**Fig 4:**
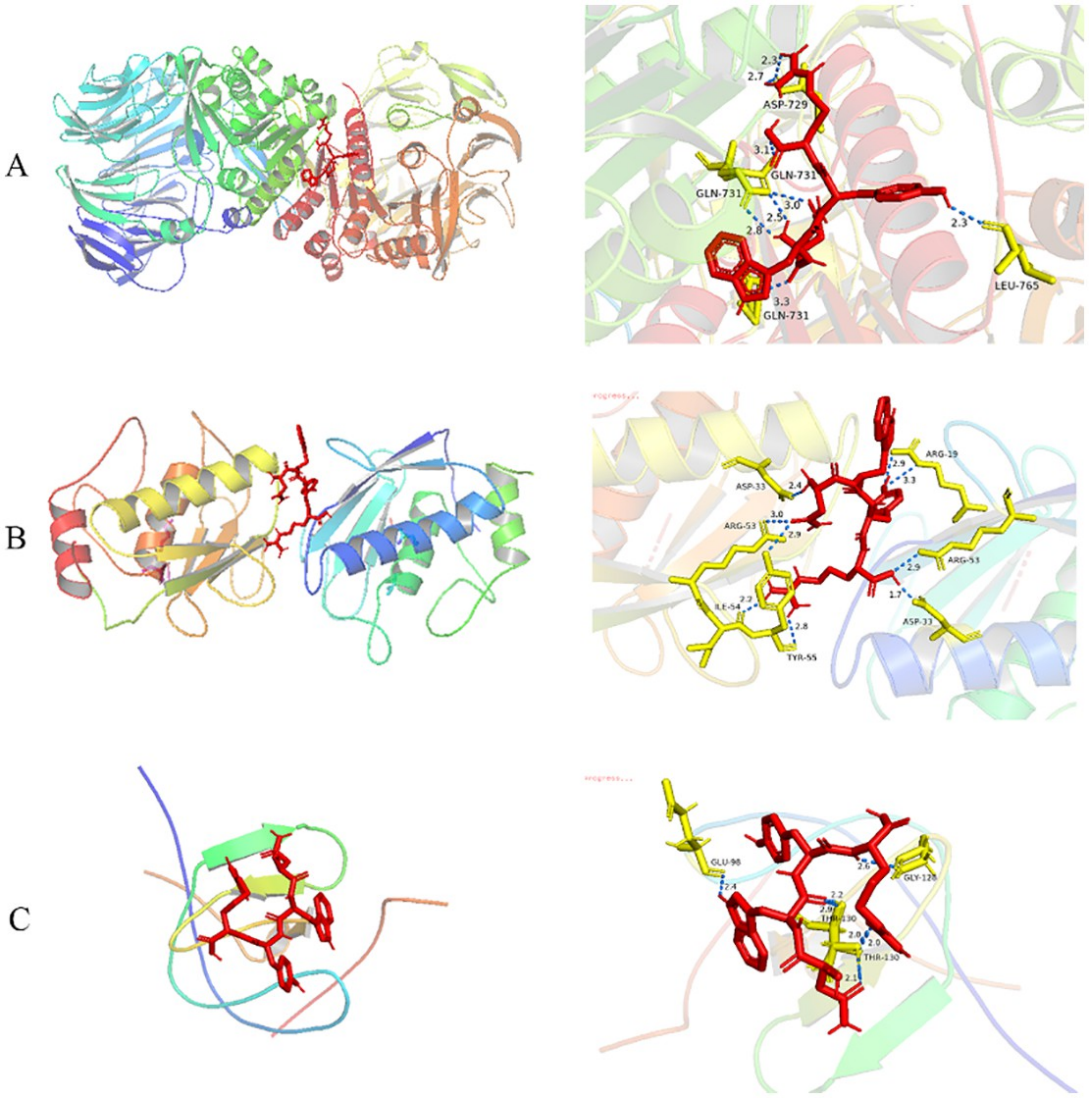
Docking schematic diagram of core targets and NWYR (A: DPP4-NWYR; B: MMP2-NWYR; C: ACE-NWYR).

Molecular docking is applied to predict the binding modes of proteins and ligands in three-dimensional structures and is widely used in structural molecular biology. Numerous studies have applied molecular docking methods to investigate the interactions between receptors and various ligands [33]. AutoDock Vina is a commonly used molecular docking program [49]. The results of molecular docking showed that the binding energy of all combinations was less than 0 kcal/mol, and proteins could spontaneously bond with small molecules. According to the structure-activity relationship analysis, DPP4 was found to interact with NWYR through eight hydrogen bonds, with bond distances ranging between 2.3 Å to 3.3 Å. The major binding site residues involved in the hydrogen bond interactions were determined to be Asp-729, Gln-731, and Leu-765 (Fig 4A). The docking results of MMP2 and NWYR showed nine hydrogen bond interactions with bond distances between 1.7 Å and 3.3 Å. These interactions emerged between hydrogen bonds and the amino acid residues Asp-33, Arg-53, Ile-54, Arg-19, and Tyr-55 (Fig 4B). Moreover, ACE and NWYR connected at Glu-98, Gly-128, and Thr-130 through hydrogen bonds, forming a total of seven hydrogen bonds with an average bond distance of 2.3 Å (Fig 4C). By molecular docking calculations, the average binding free energy values of the above proteins were -8.51 kcal/mol, -7.83 kcal/mol, and -8.1 kcal/mol, separately. We concluded that the interaction between DPP4 and NWYR is the most stable. NWYR is the optimal core target for the treatment of T2DM. Its grid size (XYZ point) is 100.0, 126.0, and 116.0, with the grid center designated as (x, y, and z) 24.022, 40.437, and 68.159. The docking results show that bioactive peptide (NWYR) mainly binds to target proteins through hydrogen bonding and binds to various amino acid residues, thereby affecting the role of the target in each pathway and achieving the purpose of improving T2DM.

## 5. Conclusions

In summary, the bioinformatics platform can identify biological peptides *in silico* hydrolysates, and porcine collagen proteins are suitable materials for the production of α-glucosidase inhibitory peptides. Our study (1) identified a novel natural peptide (NWYR) with good water solubility, high biological activity, and good ADMET properties that produces few or no side effects. (2) Through network pharmacological screening, a total of 32 common targets of NWYR and type 2 diabetes were identified, and 5 core targets were selected according to the threshold. (3) Enrichment analysis showed that NWYR regulates a variety of biological processes and molecular functions of different cellular components, and can play a hypoglycemic role through involvement in diabetic cardiomyopathy and IL-17 signaling pathways. (4) Molecular docking showed that NWYR mainly binds to target proteins through hydrogen bonding and binds to a variety of amino acid residues, thereby affecting the role of the target in the pathway and achieving the purpose of improving hyperglycemia. In conclusion, this study revealed the potential target and mechanism of action of active peptide (NWYR) in improving T2DM. Porcine collagen can be used as a suitable raw material for the preparation of hypoglycemic peptide, providing a theoretical basis for the development of NWYR as a potential hypoglycemic drug. However, in subsequent studies, it is necessary to further verify the hypoglycemic ability of porcine collagen active peptide (NWYR), and explore the hypoglycemic mechanism of NWYR from multiple perspectives such as key target genes, protein expression levels and differences in metabolites in combination with cell models and animal models, which will provide more in-depth theoretical support for its improvement in the treatment of diabetes.

## References

[1] Shen X.; Zhang M.; Bhandari B.; Gao Z. Novel technologies in utilization of byproducts of animal food processing: A review. *Critical Reviews in Food Science and Nutrition*. 2019, 59(21), 3420–3430. doi: 10.1080/10408398.2018.1493428 30285468

[2] Cao C.; Xiao Z.; Ge C.; Wu Y. Animal by-products collagen and derived peptide, as important components of innovative sustainable food systems—A comprehensive review. *Critical Reviews in Food Science and Nutrition*. 2022, 62(31), 8703–8727. doi: 10.1080/10408398.2021.1931807 34080446

[3] Kęska P.; Stadnik J. Porcine myofibrillar proteins as potential precursors of bioactive peptides–An *in silico* study. *Food & Function*. 2016, 7(6), 2878–2885. 27247979 10.1039/c5fo01631b

[4] Zhang Z.; Wang Y.M.; Qiu Y.T.; Chi C.F.; Luo H.Y.; Wang B. Gelatin From Cartilage of Siberian Sturgeon (Acipenser baerii): Preparation, Characterization, and Protective Function on Ultraviolet-A-Injured Human Skin Fibroblasts. *Frontiers in Marine Science*. 2022, 9, 925407.

[5] Wang J.; Wu T.; Fang L.; Liu C.L.; Liu X.T.; Li H.M.; et al. Peptides from walnut (Juglans mandshurica Maxim.) protect hepatic HepG2 cells from high glucose-induced insulin resistance and oxidative stress. *Food & function*. 2020, 11(9), 8112–8121. doi: 10.1039/d0fo01753a 32857071

[6] Liu L.L.; Zhang J.H.; Cheng Y., Zhu M., Xiao Z.F.; Ruan G.C.; et al. Gut microbiota: A new target for T2DM prevention and treatment. *Frontiers in Endocrinology*. 2022, 13:958218. doi: 10.3389/fendo.2022.958218 36034447 PMC9402911

[7] Grunberger G. Should side effects influence the selection of antidiabetic therapies in type 2 diabetes? *Current Diabetes Reports*. 2017, 17, 1–12. 28293908 10.1007/s11892-017-0853-8

[8] Harsch I. A., Kaestner R. H., Konturek P. C. Hypoglycemic side effects of sulfonylureas and repaglinide in ageing patients-knowledge and self-management. *Journal of Physiology & Pharmacology An Official Journal of the Polish Physiological Society*. 2018, 69(4), 647–649. doi: 10.26402/jpp.2018.4.15 30552308

[9] Sun K.L.; Gao M., Wang Y.Z.; Li X.R.; Wang P.; et al. Antioxidant Peptides From Protein Hydrolysate of Marine Red Algae Eucheuma cottonii: Preparation, Identification, and Cytoprotective Mechanisms on H2O2 Oxidative Damaged HUVECs. *Frontiers in Microbiology*. 2022, 13, 791248–791248. doi: 10.3389/fmicb.2022.791248 35531284 PMC9069057

[10] Suo S.K.; Zheng S.L.; Chi C.F.; Luo H.Y.; Wang B. Novel angiotensin-converting enzyme inhibitory peptides from tuna byproducts—milts: Preparation, characterization, molecular docking study, and antioxidant function on H2O2-damaged human umbilical vein endothelial cells. *Frontiers in Nutrition*. 2022, 9, 957778–957778. doi: 10.3389/fnut.2022.957778 35938100 PMC9355146

[11] Sheng Y.; Wang W.Y.; Wu M.F.; Wang Y.M.; Zhu W.Y.; Chi C.F.; et al. Eighteen Novel Bioactive Peptides from Monkfish (Lophius litulon) Swim Bladders: Production, Identification, Antioxidant Activity, and Stability. *Marine Drugs*. 2023, 21(3), 169. doi: 10.3390/md21030169 36976218 PMC10054418

[12] Cai W.W.; Hu X.M.; Wang Y.M.; Chi C.F.; Wang B. Bioactive Peptides from Skipjack Tuna Cardiac Arterial Bulbs: Preparation, Identification, Antioxidant Activity, and Stability against Thermal, pH, and Simulated Gastrointestinal Digestion Treatments. *Marine Drugs*. 2022, 20(10), 626–626. doi: 10.3390/md20100626 36286450 PMC9604775

[13] Kong J.; Hu X.M; Cai W.W; Wang Y.M.; Chi C.F.; Wang B. Bioactive Peptides from Skipjack Tuna Cardiac Arterial Bulbs (II): Protective Function on UVB-Irradiated HaCaT Cells through Antioxidant and Anti-Apoptotic Mechanisms. *Marine Drugs*. 2023, 21(2), 105–105. doi: 10.3390/md21020105 36827146 PMC9962892

[14] Salim M.A.S.M.; Gan C.Y. Dual-function peptides derived from egg white ovalbumin: Bioinformatics identification with validation using in vitro assay. *Journal of functional foods*. 2020, 64, 103618.

[15] Nong N.T.P.; Chen Y.K.; Shih W.L.; Hsu J.L. Characterization of novel dipeptidyl peptidase-iv inhibitory peptides from soft-shelled turtle yolk hydrolysate using orthogonal bioassay-guided fractionations coupled with *In Vitro* and *In Silico* study. *Pharmaceuticals*. 2020, 13(10), 308. 33066488 10.3390/ph13100308 PMC7602288

[16] Jin R.; Teng X.; Shang J.; Wang D.; Liu N. Identification of novel DPP–IV inhibitory peptides from Atlantic salmon (Salmo salar) skin. *Food Research International*. 2020, 133, 109161. doi: 10.1016/j.foodres.2020.109161 32466942

[17] Gao J.; Gong H.,; Mao, X. Dipeptidyl peptidase-IV inhibitory activity and related molecular mechanism of bovine α-lactalbumin-derived peptides. *Molecules*. 2020, 25(13), 3009. 32630113 10.3390/molecules25133009 PMC7412263

[18] Xiang X.; Lang M.; Li Y.; Zhao X.; Sun H.M.; Jiang W.W.; et al. Purification, identification and molecular mechanism of dipeptidyl peptidase IV inhibitory peptides from discarded shrimp (Penaeus vannamei) head. *Journal of Chromatography B*. 2021, 1186, 122990. doi: 10.1016/j.jchromb.2021.122990 34735973

[19] Federico S.; Nöchel U.; Löwenberg C.; Lendlein A.; Neffe A.T. Supramolecular hydrogel networks formed by molecular recognition of collagen and a peptide grafted to hyaluronic acid. *Acta biomaterialia*. 2016, 38, 1–10. doi: 10.1016/j.actbio.2016.04.018 27090592

[20] Nong N. T. P.; Hsu J. L. Characteristics of food protein-derived antidiabetic bioactive peptides: A literature update. *International Journal of Molecular Sciences*. 2021, 22(17), 9508. doi: 10.3390/ijms22179508 34502417 PMC8431147

[21] Devasia S.; Kumar S.; Stephena P.S.; Inoue N.; Sugihara F.; Suzuki K. Double blind, randomized clinical study to evaluate efficacy of collagen peptide as add on nutritional supplement in Type 2 diabetes. *Journal of Clinical Nutrition and Food Science*. 2018, 6–11.

[22] Dowarah J.; Singh V.P. Anti-diabetic drugs recent approaches and advancements. *Bioorganic & medicinal chemistry*. 2020, 28(5), 115263. doi: 10.1016/j.bmc.2019.115263 32008883

[23] Mojica L.; De Mejía E.G. Optimization of enzymatic production of anti-diabetic peptides from black bean (Phaseolus vulgaris L.) proteins, their characterization and biological potential. *Food & function*. 2016, 7(2), 713–727. doi: 10.1039/c5fo01204j 26824775

[24] Valencia-Mejía E.; Batista K.A.; Fernández J.J. A.; Fernandes K.F. Antihyperglycemic and hypoglycemic activity of naturally occurring peptides and protein hydrolysates from easy-to-cook and hard-to-cook beans (Phaseolus vulgaris L.). *Food Research International*. 2019, 121, 238–246. doi: 10.1016/j.foodres.2019.03.043 31108745

[25] Zhang Y.; Chen R.; Zuo F.; Ma H.; Zhang Y.; Chen S. Comparison of dipeptidyl peptidase IV-inhibitory activity of peptides from bovine and caprine milk casein by *in silico* and *in vitro* analyses. *International Dairy Journal*. 2016, 53, 37–44.

[26] He L.; Wang X.Y.; Wang Y., Luo J.; Zhao Y.N.; Han G.X.; et al. Production and identification of dipeptidyl peptidase IV (DPP-IV) inhibitory peptides from discarded cowhide collagen. *Food Chemistry*. 2023, 405, 134793. doi: 10.1016/j.foodchem.2022.134793 36335727

[27] Li, D.J.; Wang, D.H.; Yan, S.K. Exploration of underlying molecular mechanism of Lycii Cortex in Treating Type 2 Diabetes Mellitus Based on Network Pharmacology and Molecular Docking. *In E3S Web of Conferences*. EDP Sciences. 2021, 233: 02007.

[28] Pan RY, Ren GY, Ma FL, Study on hypoglycemic mechanism of aloe emodin based on network pharmacology and molecular docking [J/OL]. *Food and fermentation industry*: 1–10[2023-10-11]

[29] Zhou M.; Ren G.Y.; Zhang B.; Ma F.L.; Fan J.L.; Qiu Z.J. Screening and identification of a novel antidiabetic peptide from collagen hydrolysates of Chinese giant salamander skin: network pharmacology, inhibition kinetics and protection of IR-HepG2 cells. *Food & Function*. 2022, 13(6), 3329–3342. doi: 10.1039/d1fo03527d 35260876

[30] Tian W, Li X M, Yang J, et al. Study on bacteriostatic active components of Radix isatidis and their mechanism of action based on network pharmacology [J]. *Acta Veterinaria Sinica*, 2022, 53 (08): 2782–2793.

[31] Minkiewicz P.; Iwaniak A.; Darewicz M. BIOPEP-UWM database of bioactive peptides: Current opportunities. *International journal of molecular sciences*. 2019, 20(23), 5978. doi: 10.3390/ijms20235978 31783634 PMC6928608

[32] Duvaud S.; Gabella C.; Lisacek F.; Stockinger H.; Ioannidis V.; Durinx C. Expasy, the Swiss Bioinformatics Resource Portal, as designed by its users. *Nucleic Acids Research*. 2021, 49(W1), W216–W227. doi: 10.1093/nar/gkab225 33849055 PMC8265094

[33] Chen J.B.; Yu X.D.; Chen Q.Z.; Wu Q.Y.; He Q.Y. Screening and mechanisms of novel angiotensin-I-converting enzyme inhibitory peptides from rabbit meat proteins: A combined *in silico* and *in vitro* study. *Food Chemistry*. 2022, 370, 131070. 34537424 10.1016/j.foodchem.2021.131070

[34] Cheng F.X.; Li W.H.; Zhou Y.D.; Shen J.; Wu Z.R.; Liu G.X.; et al. Correction to “admetSAR: A Comprehensive Source and Free Tool for Assessment of Chemical ADMET Properties”. *Journal of Chemical Information and Modeling*. 2019, 59(11), 4959–4959. 31661262 10.1021/acs.jcim.9b00969

[35] Ramadhan A.H.; Nawas T., Zhang X.W.; Pembe W.M.; Xia W.S.; Xu Y.S. Purification and identification of a novel antidiabetic peptide from Chinese giant salamander (Andrias davidianus) protein hydrolysate against α-amylase and α-glucosidase. *International journal of food properties*. 2017, 20(sup3), S3360–S3372.

[36] Szklarczyk D.; Gable A.L.; Nastou K.C.; Lyon D.; Kirsch R.; Pyysalo S.; et al. The STRING database in 2021: customizable protein–protein networks, and functional characterization of user-uploaded gene/measurement sets. *Nucleic acids research*. 2021, 49(D1), D605–D612. doi: 10.1093/nar/gkaa1074 33237311 PMC7779004

[37] Zhang J.X.; Chen Z.Y.; Zhang L.L.; Zhao X.X.; Liu Z.G.; Zhou W. A systems-based analysis to explore the multiple mechanisms of Shan Zha for treating human diseases. *Food & Function*. 2021, 12(3), 1176–1191. 33432314 10.1039/d0fo02433c

[38] Kuzniak-Glanowska E.; Glanowski M.; Kurczab R.; Bojarski A. J.; Podgajny R. Mining anion–aromatic interactions in the Protein Data Bank. *Chemical Science*. 2022, 13(14), 3984–3998. doi: 10.1039/d2sc00763k 35440982 PMC8985504

[39] Bhandari D.; Rafiq S.; Gat Y.; Gat P.; Waghmare R.; Kumar V. A review on bioactive peptides: Physiological functions, bioavailability and safety. *International Journal of Peptide Research and Therapeutics*. 2020, 26, 139–150.

[40] Chakrabarti S.; Guha S.; Majumder K. Food-derived bioactive peptides in human health: Challenges and opportunities. *Nutrients*. 2018, 10(11), 1738. doi: 10.3390/nu10111738 30424533 PMC6265732

[41] Zheng S.L.; Wang Y.Z.; Zhao Y.Q.; Chi C.F.; Zhu W.Y.; Wang B. High Fischer ratio oligopeptides from hard-shelled mussel: Preparation and hepatoprotective effect against acetaminophen-induced liver injury in mice. *Food Bioscience*. 2023, 53, 102638.

[42] Toldrá F.; Reig M.; Aristoy M.C.; Mora L. Generation of bioactive peptides during food processing. *Food chemistry*. 2018, 267, 395–404. doi: 10.1016/j.foodchem.2017.06.119 29934183

[43] Martinez-Mayorga K.; Madariaga-Mazon A.; Medina-Franco J.L.; Maggiora G. The impact of chemoinformatics on drug discovery in the pharmaceutical industry. *Expert Opinion on Drug Discovery*. 2020, 15(2), 1–14. doi: 10.1080/17460441.2020.1696307 31965870

[44] Naveja J.J.; Rico-Hidalgo M.P.; Medina-Franco J.L. Analysis of a large food chemical database: chemical space, diversity, and complexity. *F1000 Research*. 2018, 7, 993. doi: 10.12688/f1000research.15440.2 30135721 PMC6081979

[45] Fan Y., Yu Z.P.; Zhao W.Z.; Ding L., Zheng F.P.; Li J.R.; et al. Identification and molecular mechanism of angiotensin-converting enzyme inhibitory peptides from Larimichthys crocea titin. *Food Science and Human Wellness*. 2020, 9(3), 257–263.

[46] Han L.; Zhang L.L.; Ma W.F.; Li D.; Shi R.J.; Wang M. Proanthocyanidin B2 attenuates postprandial blood glucose and its inhibitory effect on alpha-glucosidase: analysis by kinetics, fluorescence spectroscopy, atomic force microscopy and molecular docking. *Food & function*. 2018, 9(9), 4673–4682. doi: 10.1039/c8fo00993g 30188554

[47] Tao S.N.; Chen G.J.; Xu W.Q.; Peng Y.J.; Wan P.; Sun Y.; et al. Preparation of theasinensin A and theasinensin B and exploration of their inhibitory mechanism on α-glucosidase. *Food & function*. 2020, 11(4), 3527–3538. 32255112 10.1039/c9fo03054a

[48] Fang R.Z.; Meng Y.Y.; Tang X.S.; Li X.F.; Wang X.P. Study on Anti-Type 2 Diabetes Mechanism of Sophora japonica L. based on Network Pharmacology and Molecular Docking. *Medicine and health*. 2021, 10(4).

[49] Wang Z.; Sun H.Y.; Yao X.J.; Li D.; Xu L.; Li Y.Y.; et al. Comprehensive evaluation of ten docking programs on a diverse set of protein–ligand complexes: the prediction accuracy of sampling power and scoring power. *Physical Chemistry Chemical Physics*. 2016, 18(18), 12964–12975. doi: 10.1039/c6cp01555g 27108770

